# Comparison of In-Vivo and Ex-Vivo Ascending Aorta Elastic Properties through Automatic Deep Learning Segmentation of Cine-MRI and Biomechanical Testing

**DOI:** 10.3390/jcm12020402

**Published:** 2023-01-04

**Authors:** Emmanouil Markodimitrakis, Siyu Lin, Emmanouil Koutoulakis, Diana Marcela Marín-Castrillón, Francisco Aarón Tovar Sáez, Sarah Leclerc, Chloé Bernard, Arnaud Boucher, Benoit Presles, Olivier Bouchot, Thomas Decourselle, Marie-Catherine Morgant, Alain Lalande

**Affiliations:** 1 ImViA Laboratory, EA 7535, University of Burgundy and Franche-Comte, 21000 Dijon, France; 2 Department of Cardio-Vascular and Thoracic Surgery, University Hospital of Dijon, 21079 Dijon, France; 3 CASIS—CArdiac Simulation & Imaging Software SAS, 21800 Quetigny, France; 4 Department of Medical Imaging, University Hospital of Dijon, 21079 Dijon, France

**Keywords:** cine-MRI, deep learning segmentation, ascending aortic elasticity, young modulus

## Abstract

Ascending aortic aneurysm is a pathology that is important to be supervised and treated. During the years the aorta dilates, it becomes stiff, and its elastic properties decrease. In some cases, the aortic wall can rupture leading to aortic dissection with a high mortality rate. The main reference standard to measure when the patient needs to undertake surgery is the aortic diameter. However, the aortic diameter was shown not to be sufficient to predict aortic dissection, implying other characteristics should be considered. Therefore, the main objective of this work is to assess in-vivo the elastic properties of four different quadrants of the ascending aorta and compare the results with equivalent properties obtained ex-vivo. The database consists of 73 cine-MRI sequences of thoracic aorta acquired in axial orientation at the level of the pulmonary trunk. All the patients have dilated aorta and surgery is required. The exams were acquired just prior to surgery, each consisting of 30 slices on average across the cardiac cycle. Multiple deep learning architectures have been explored with different hyperparameters and settings to automatically segment the contour of the aorta on each image and then automatically calculate the aortic compliance. A semantic segmentation U-Net network outperforms the rest explored networks with a Dice score of 98.09% (±0.96%) and a Hausdorff distance of 4.88 mm (±1.70 mm). Local aortic compliance and local aortic wall strain were calculated from the segmented surfaces for each quadrant and then compared with elastic properties obtained ex-vivo. Good agreement was observed between Young’s modulus and in-vivo strain. Our results suggest that the lateral and posterior quadrants are the stiffest. In contrast, the medial and anterior quadrants have the lowest aortic stiffness. The in-vivo stiffness tendency agrees with the values obtained ex-vivo. We can conclude that our automatic segmentation method is robust and compatible with clinical practice (thanks to a graphical user interface), while the in-vivo elastic properties are reliable and compatible with the ex-vivo ones.

## 1. Introduction

The aorta is the largest artery in the human body that performs the critical task of supplying oxygen-rich blood and nutrients to vital organs and major arteries. If disease or injury affects blood flow through this vessel, life-threatening complications can occur in minutes [1,2]. A serious condition where the thoracic aorta progressively dilates even fifty percent more than its average diameter is defined as an ascending aortic aneurysm, leading the aortic wall to become weakened and stiff, while its elastic properties decrease. Although it may occur in young people, this condition is more common in older adults [3].

Aortic dilation can lead to aortic dissection, which threatens to tear apart (rupture) the inner aortic wall [4]. Aortic dissection can lead to internal bleeding, implying the blood can flow on two channels instead of one. The ascending aorta dissection (type A, Stanford classification [5]) is associated with high levels of mortality and needs immediate intervention.

Current guidelines are based on the maximum diameter of the ascending aorta and recommend prophylactic replacement if the diameter exceeds 55 mm [6]. However, there are some tissue disorders where the diameter threshold should be lower than this threshold value. Some studies [7,8] emerge that in many cases of acute aortic dissection, the diameter is less than 55 mm. Lin et al. [9] declared that there is a need for new, more effective criteria for the prevention of aneurysm complications based on biaxial tensile tests on the human ascending aorta. For instance, we can consider parameters like aortic compliance and Young’s modulus [10] that quantify the biomechanical properties of the aorta. Many connective tissue disorders (such as Marfan syndrome, Elher-Danlos syndrome, Loeys-Detz syndrome, and MYH11 mutation) can be studied based on the evaluation of aortic elasticity [11].

Morgant et al. [12] compared global ex-vivo elastic properties of the aorta with in-vivo ones. Their results imply that a high correlation exists between the aortic stiffness measured by the maximum Young’s modulus and the aortic compliance measured from magnetic resonance imaging (MRI). Although those results are inefficient in proving that aortic compliance is a criterion for operative indication, they proved the robustness of cardiac-MRI (CMRI) imaging in assessing the elastic properties of the aorta.

Concerning the in-vivo evaluation, MRI is a well-established, rapidly evolving field of cardiovascular medical imaging [13]. Nowadays, there are two simple potential in-vivo approaches based on MRI acquisitions to assess the parameters of the aortic elasticity. Either by measuring the pulse wave velocity [14] which describes the overall (or global) stiffness of the aorta or by studying the aortic compliance, which describes locally the dynamic expansion of the aortic wall across the cardiac cycle [15]. This study concentrates on the second approach, assessing aortic compliance, defined as the cross-sectional area variation during a cardiac cycle divided by the pulse pressure variation. While studies like the one of Morgant et al. [12] assessed the global elastic properties of the aorta, our study contribution is based on the in-vivo assessment of local biomechanical properties. The reason we study the local properties is that the aortic wall certainly involves differences in the behavior of stiffness depending on the localization [9]. Considering four different quadrants, each one should result in different elasticity and distensibility properties. Considering this ex-vivo study, the lateral quadrant was the stiffest, while the medial quadrant had the lowest aortic stiffness across the walls. Local distensibility measurements from ex-vivo experiments [9,12] named maximum Young’s modulus and physiological Young’s modulus are compared with local in-vivo compliance of each quadrant to analyze the possible correlation between them. Aortic samples retrieved during surgery are stretched to investigate their ex-vivo elastic properties. To compare in-vivo and ex-vivo experiments, we also study the measurement of local strain of the aortic wall in-vivo and ex-vivo. The purpose of this comparison is to investigate if our proposed non-invasive method for the calculation of aortic elasticity is reliable. However, to calculate the cross-sectional area from two-dimensional (2D) axial cine-MRI acquisitions at the level of the pulmonary trunk, there is a need for ascending aorta localization and segmentation.

Manual segmentation of the aorta is a time-consuming task that introduces intra-observer and inter-observer variabilities and bias error [16]. Then, automation of the segmentation process is necessary. One of the primary objectives of our study is to provide a robust automated technique for two-dimensional ascending aorta segmentation from cine-MRI at the level of the pulmonary trunk using deep learning (DL) image processing methods. Deep learning is a subcategory of machine learning and artificial intelligence which has many breakthroughs in image analysis and processing. A robust and stable DL method is proposed to segment the ascending aorta from cine-MRI in axial orientation at the level of the pulmonary trunk. Multiple architectures are trained on clinical data, and the most accurate one has been selected.

To study the variability of elasticity and distensibility among the aortic area, the segmented aorta was positioned into four quadrants relative to the medial, posterior, lateral, and anterior areas according to the aortic wall. The evaluation of our method is divided into two parts. The first one is the evaluation between aorta segmentation predicted results of the DL model and ground truth provided by a medical expert, using metrics like Dice coefficient, Intersection over Union (IoU), and Hausdorff distance, precision, and recall. The second part is based on the aortic wall’s elastic properties and a comparison between in-vivo and ex-vivo results [9,12]. Then our work focused on the evaluation of the global and local elastic properties of the aorta (compliance and strain) and compared them with ex-vivo biomechanical properties. All of the above objectives are incorporated into a user-friendly graphical user interface.

## 2. Materials and Methods

The study population consisted of 73 patients. All patients had an aneurysm of the ascending aorta, and surgery was required. Patients with connective tissue disease were not included in our study group. MRI acquisition was performed pre-operatively. The acquisition protocol included a sequence of images specifically for the evaluation of aortic compliance. On average, 30 slices of cine-MRI were acquired for each patient. The patient’s blood pressure was recorded during the exam and used to calculate the aortic compliance.

A deep learning model was trained to segment the ascending aorta automatically. The aortic compliance was calculated based on the segmented aorta. In parallel, for comparison, a medical expert performed semi-automatic contouring of the ascending aorta using the commercial software QIR (CASIS, Quetigny, France). Using the center of gravity of the aorta, we automatically divided the aortic surface into four quadrants to calculate the local elastic properties.

For the ex-vivo evaluation, the collected aortic wall samples from the replacement procedure were partitioned relative to medial, posterior, lateral, and anterior quadrants. The quadrants of the aorta were stretched biaxially until rupture and Young’s modulus was calculated. This parameter measures the ability of a material to withstand changes in length (strain) when under lengthwise tension or compression (tensile stress).

We compared the results of the in-vivo and ex-vivo experiments, leveraging common patients between the datasets. The compared values are in-vivo compliance, strain, and ex-vivo Young’s modulus and strain.

### 2.1. Related Work

Most of the published studies concerning aorta segmentation in the same framework are implemented with classical image processing techniques and semi-automated methods. Most authors consider the whole cardiac cycle, using fully or semi-automated but not deep learning methods, allowing the creation of cross-sectional area vs time curves [16,17,18,19]. Likewise, Miteran et al. [20] detect the aortic wall using an adaptation of a curvilinear region detector. Following the segmentation, they get the maximum and minimum areas without manual selection of the systolic phase, but this approach is not free of errors [7,8]. Although some of those methods derive promising results, most are semi-automated, requiring user input (for example, the center of the aorta). From the methods mentioned above, only the work of Miteran et al. [20] is fully automated, it is however not a DL-based implementation. Thus, we conclude there is a lack of fully automated methods, for which DL is a strong but unexplored candidate.

### 2.2. Clinical Background

The formulas for the calculation of the aortic elastic properties are defined in this subsection. Specifically, aortic compliance, Young’s modulus, and strain seem to be relevant for the evaluation of aortic elasticity.

#### 2.2.1. Aortic Compliance

A measure to describe the elasticity of the aortic walls is aortic compliance, defined as the cross-sectional area variation during a cardiac cycle divided by the pulse pressure. The mathematical formula is specified in the Equation (1). In this study, the cross-sectional area is calculated based on 2D axial thoracic MRI slices at the level of the pulmonary trunk along the cardiac cycle. The pressure is acquired during the MRI acquisition.

(1) $$Compliance=\frac{MaximumArea-MinimumArea}{SystolicPressure-DiastolicPressure}\left(\frac{mm^{2}}{mmHg}\right)$$

Moreover, local aortic compliance is studied, where we divide the cross-sectional area into four quadrants based on the center of gravity of the aorta. The compliance of each quadrant is named local compliance.

#### 2.2.2. Young’s Modulus and Strain

Young’s modulus is a metric for determining how difficult it is to produce elastic deformation in solid materials. Applying a load *F* to elastic materials can result in a change in length *l*. The stress σ is the external force perpendicular to the cross-sectional area *A*, and the engineering strain ε is the length variation in its vertical direction. The force and the length determine the stresses and strains.

Engineering strain is defined as the ratio of the resting specimen length (after preconditioning) $l_{0}$ divided by the deformed length Δl, where Δl is the difference in length between the resting and load-filled specimens.

(2) $$\varepsilon =\frac{l-l_{0}}{l_{0}}=\frac{\Delta l}{l_{0}}$$

The amount of tensile load *F* recorded during the test per unit loaded cross-sectional area *A* of the specimen is referred to as stress σ [21]. Aortic tissue is considered incompressible tissue. Area *A* is equal to the cross-sectional area of the resting specimen $A_{0}$, where $A_{0}$ is calculated using the load-free specimen thickness and length $l_{0}$.

Stress σ can be computed as:

(3) $$\sigma =\frac{F}{A_{0}}$$

The elastic modulus, also known as Young’s modulus, is a widely used term to describe the biomechanical properties of human tissue [22,23,24,25]. Young’s modulus was calculated for the evaluation of the sample stiffness [26]. The calculation of Young’s modulus in aortic tissue can be done in biaxial tensile tests [27,28,29]. Young’s modulus at various stress levels can be calculated as the stress-strain curve’s first derivative of stress overstretch such as:

(4) $$E=\frac{\sigma }{\varepsilon }$$

The physiological range of Young’s modulus was determined by the patient’s blood pressure. The maximum value of Young’s modulus was identified by the maximum ratio of the stress and strain curve [9].

To calculate in-vivo strain from MR images, we consider the perimeter of the aorta (defined by the aortic wall). Strain is defined as the difference between the maximum and minimum perimeter divided by the minimum perimeter:

(5) $$Strain=\frac{MaximumPerimeter-MinimumPerimeter}{MinimumPerimeter}$$

### 2.3. Data

#### 2.3.1. In-Vivo Data: Magnetic Resonance Imaging (MRI)

**Figure 1:**
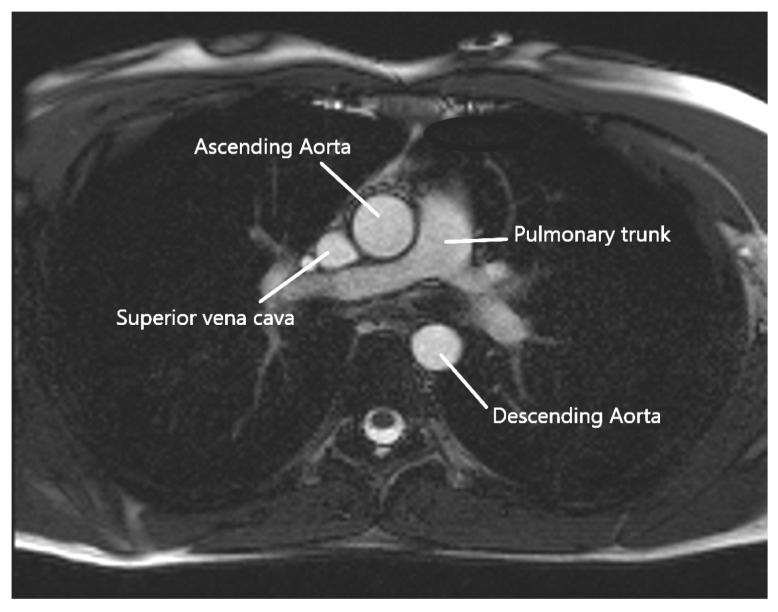
Axial MRI at the level of the pulmonary trunk.

A cardiac MRI was performed before the cardiac surgery to determine, in particular, the aortic compliance. Images were collected using a 3T MRI (Skyra, Siemens Healthineers, Erlangen, Germany), with a specific acquisition in addition to the standard protocol. This acquisition is a FLASH-type sequence undertaken during a short breath-hold in the transverse plane at the level of the pulmonary artery bifurcation, as shown in Figure 1. Since it is less sensitive to noise caused by rapid or turbulent flow at 3T, this sequence was chosen over a steady state free precession (SSFP)–type sequence. This plane allows us to examine both the ascending and descending thoracic aortas. Images at all phases of the cardiac cycle were obtained with a temporal resolution of 20 ms to 34 ms thanks to a retrospective ECG-gating, with the following sequence settings: echo time of 3.42 ms, repetition time of 7.21 ms, flip angle of 12°, spatial resolution between 1.09 × 1.09 mm${}^{2}$/pixel, and 1.25 × 1.25 mm${}^{2}$/pixel (corresponding to a field of view ranging from 350 mm to 400 mm), and slice thickness of 5 mm. A generalized auto-calibrating partially parallel acquisitions (GRAPPA) was performed, with an acceleration factor of two. A pre-scan normalized filter and distortion correction were also applied.

The dataset consists of 73 exams, with an average of 30 slices per case across the cardiac cycle, concluding in a total of 2189 slices. Each patient’s systolic and diastolic pressures have been recorded during the exam. The ground truth masks have been segmented by an expert using the semi-automated QIR software (Casis, Quetigny, France). The software proposes a potential segmentation and the medical expert has to correct it precisely if necessary.

**Figure 2:**
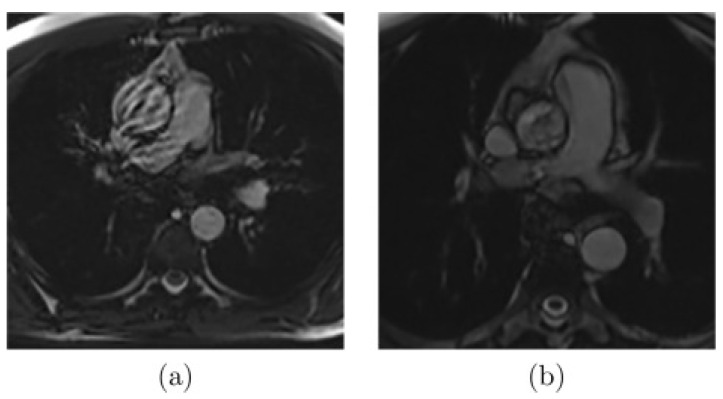
Examples of MR images with artifacts due to (**a**) rapid or (**b**) turbulent blood flow [20].

**Figure 3:**
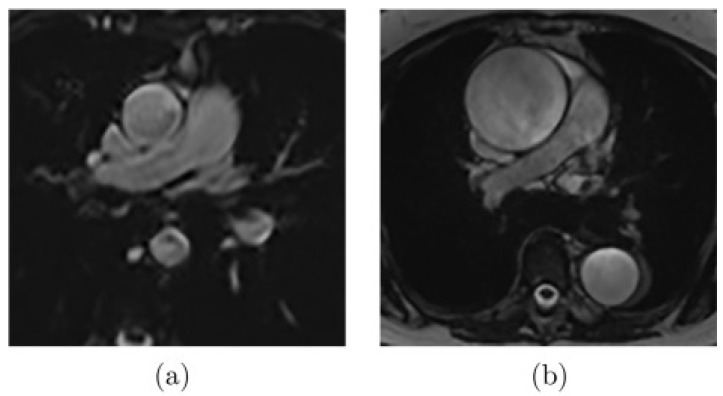
Examples of MR images where (**a**) the aortic wall is not well-defined due to the influence of the proximity of other structures or (**b**) the aorta is highly dilated [20].

As presented in Figure 2, there are images where noise or artifacts are often present in the critical part of the cardiac cycle, especially for the ascending aorta, as the FLASH sequence is sensitive to rapid and turbulent flow. Another extreme case that complicates the segmentation (Figure 3a) appears when the aortic border is poorly visible due to the proximity of some other structures. Finally, other common difficulties can be encountered, such as the highly dilated ascending aorta as shown in Figure 3b.

The dataset was split into training and test sets consisting respectively of 1784 and 405 images to train and evaluate different deep learning architectures. A validation set is not necessary for this work because all the training experiments have been implemented with K-fold cross-validation [30].

#### 2.3.2. Ex-Vivo Data

**Figure 4:**
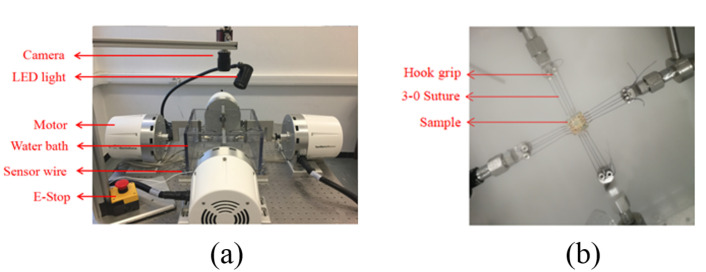
Biaxial tensile test machine. (**a**) Biaxial test bench. (**b**) The sample displacement during the test.

The aortic wall samples were preserved in phosphate-buffered saline during the transfer from the operation room to the laboratory for the tensile experiment. The samples were cut in square size (15 mm × 15 mm). The biomechanical experiments were carried out by a biaxial tensile test machine (LM1 Planar Biaxial, TA Instruments, New Castle, the United States of America) (Figure 4). Each specimen was placed in ten times of 10% preconditioning to deform the contraction caused by the sectioned aorta. Further stretching, at a rate of 10 mm·min${}^{-1}$, was programmed until rupture [9].

### 2.4. Deep Learning Segmentation and In-Vivo Elasticity Assessment

**Figure 5:**
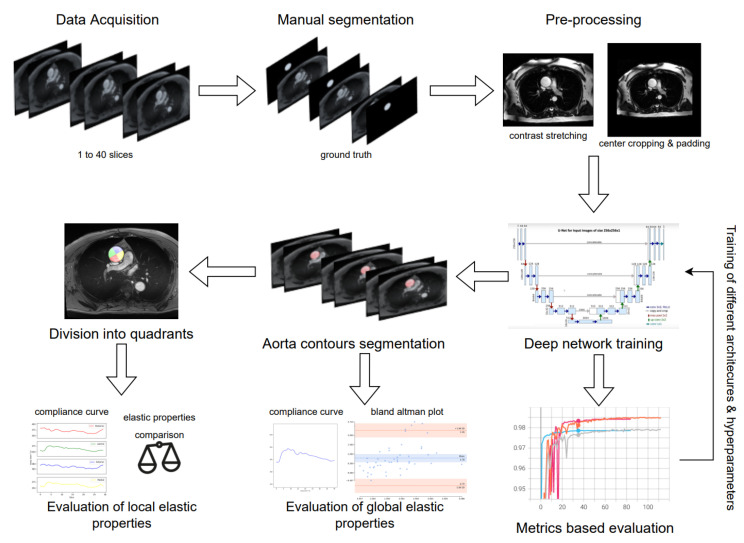
Workflow of the research strategy.

In summary, this section describes the automatic segmentation of the aorta and the in-vivo assessment of the elastic properties. The workflow is presented briefly in Figure 5. For the training of the deep learning model, multiple U-Net-based [31] architectures and hyperparameters have been explored. Some of the hyperparameters, like learning rate, had a significant impact during the training. Then the extraction of the aortic contours was obtained with the model which had the best model performance based on metrics like the Dice coefficient and Hausdorff distance. The workflow’s last stage is the automatic division of the aortic surface into four quadrants. Then, we evaluated the global and local elastic properties (compliance and strain). The elastic properties were calculated locally (for each quadrant) to explore if those properties have a link with the ex-vivo results.

#### 2.4.1. Preprocessing

A series of simple image processing techniques were performed to prepare the dataset for the training process. A min-max normalization was done to scale pixel intensity between 0 and 1. Histogram stretching was implemented, which stretches the pixel values of an image among the range of histograms. Histogram stretching can be useful to enhance image contrast, where images are difficult to comprehend because of low contrast or poor lighting. Then, a customized center-crop-padding method was used to crop the large-dimension images to the desired 256 × 256 dimensions. To handle images smaller than the required dimensions, zero padding was added around the four sides of the image. The cropping was implemented focusing on the center of the image. Using this customized method, the geometry of the image remains intact.

#### 2.4.2. Data Augmentation

Datasets with limited size can introduce overfitting and low accuracy. That is why data augmentation is necessary for some complex deep-learning tasks to improve accuracy and tackle overfitting. Random transformations on images using automatized software were performed [32]. Those transformations consist of random rotation, random horizontal and vertical flip, and random affine transformation.

#### 2.4.3. Fully Convolutional Networks and U-Net

In this section, we briefly present the U-Net architecture with the modified versions of it that we have explored and experimented with.

**Figure 6:**
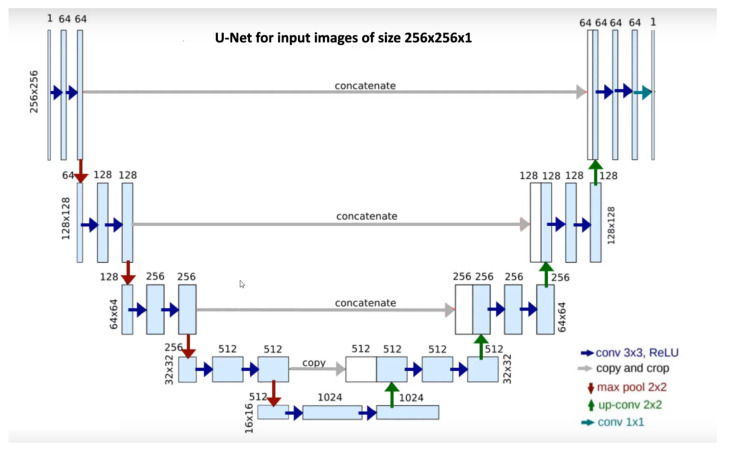
U-Net architecture used in this work [31]. Every blue box is a multichannel feature map, the number of the channel is denoted on the top of each box. The size information is provided at the lower left edge of each box. White boxes represent the feature maps that are copied through the skip connections, and the arrows represent the different operations.

U-Net [31] was designed for medical image segmentation, nevertheless, it performs excellently in a wide range of segmentation tasks across different fields. It consists of one encoder and one decoder path, in the shape of “U” shown in Figure 6 similarly with SegNet, but with one significant addition, the skip connections. The advantage of skip connections is that they provide additional higher-level spatial feature information to the decoder.

In our work, multiple experiments have been implemented with U-Net and other modified architectures of U-Net, like Residual-U-Net [33] where each sub-module of U-Net is replaced with a residual connection and a dense layer. Attention-U-Net was introduced by Oktay et al. [34], where an attention mechanism is added to readjust the encoder’s output characteristics and provide the decoder knowledge about high-level spatial information through attention gates. Attention-Residual-U-Net [35] inspired by Residual Network [36] implement both residual blocks and attention gates. Similarly, inspired by Recurrent Neural Networks (RNN), Zahangir et al. [37] propose Recurrent-Residual-U-Net, which is a combination of RNNs and residual blocks. Moreover, U-Net++ is a modification of the U-Net encoder-decoder network where the encoder and decoder sub-networks are connected through a series of nested, dense skip pathways [38]. Considering the size of the images in our dataset, input dimensions of 256 × 256 have been chosen. Our U-Net implementation has some minor modifications from the original one. On the convolutional block, after each convolutional layer and before each Rectified Linear Unit (ReLU) activation, an instance normalization layer was added.

#### 2.4.4. Training

The dataset was divided into training and testing sets. Twenty percent of the total images have been selected as the test set. For each exam, all of the slices are in the same set, either training or testing. Four different U-Net-based neural networks were trained, simple U-Net [31], Residual U-Net [33], attention U-Net [34], and attention residual U-Net [35]. During the training phase, Adaptive Moment Estimation (ADAM) was chosen as the gradient descent optimizer. This method is extremely efficient when dealing with complex data involving many information or parameters, as it uses less memory. The comparison of the models has been implemented based on metrics such as the Dice coefficient, Hausdorff distance, intersection over union (IoU), precision, and recall.

As shown in Table 1, different experiments were implemented for each architecture with various hyperparameters. For example, a static learning rate did not improve performance, so a learning rate scheduler was chosen. The scheduler is mandatory so the training does not converge to local minima. It is responsible for reducing the learning rate during the training, so different initial learning rates were tried like 0.1, 0.001, etc.

Concerning the loss function, Dice loss was selected as it outperformed the Focal and Tversky losses. Dice loss is defined as:

$$DiceLoss=1-DiceCoefficient=1-\frac{2TP}{2TP+FP+FN}$$

where *TP* refers to True Positive, *FP* to False Positive, and *FN* to False Negative pixels.

Two hundred epochs were selected, but the training process was always terminated earlier due to an early stopping strategy. As a normalization technique, instance layer normalization has been applied after each convolutional block. The training process was implemented using K-fold cross-validation with K equal to five.

The initial learning rate was set to 0.001 with a decay of 0.1 over the epochs when the validation loss had not improved for five epochs. Likewise, instance layer normalization was chosen instead of batch normalization. On average, the total training time was around 30 to 40 epochs with a maximum training process of two hours, as we implemented early stopping with patience of 16.

## 3. Results

The evaluation was implemented in three phases. Initially, we compared four different U-Net architectures, trained on a dataset of 73 patients. Then we selected the architecture with the highest performance to segment the aorta. For the second phase, we computed the aortic compliance and compared it with the ground truth. The automatically computed compliance is also compared with ex-vivo elastic properties. Finally, we computed the in-vivo strain and compared it with ex-vivo elastic properties. Between our dataset and the ex-vivo dataset [9], 22 common patients were selected with ex-vivo elastic properties.

### 3.1. Evaluation of Deep Learning Models

**Figure 7:**
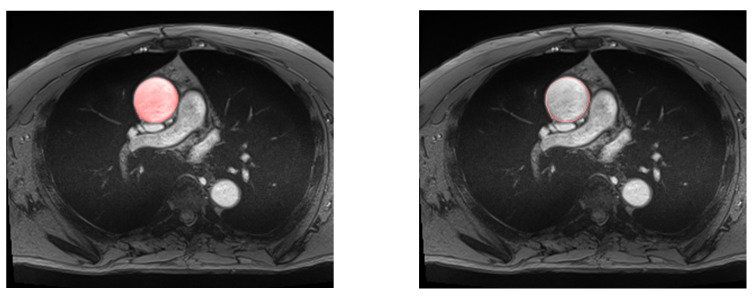
Example of automatic segmentation of the ascending aorta with our U-Net architecture. On the left, all the aortic surface is colored, in contrast to the right, where only the aortic wall is displayed.

Table 2 presents the results of all of the considered models. The performance of the simple U-Net architecture is displayed in Figure 7.

Considering that residual U-Net and simple U-Net had the highest performance, the simple U-Net was selected for the next phases as it has less complex architecture and provides a lower Hausdorff distance.

### 3.2. Global Elastic Properties’ Evaluation

To quantify the relationship between our results (predicted min-max areas and compliance) and the expected results, a statistical correlation between them is provided in Table 3. To determine if the correlation is statistically significant the p-values are also provided. Moreover, based on Bland-Altman plots [39], the average differences between the ground truth and predicted values are provided, as well as the standard deviation of that difference.

### 3.3. Local Elastic Properties’ Evaluation

**Figure 8:**
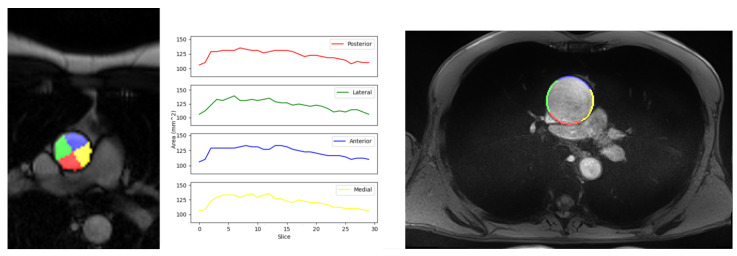
The left image is the division of the aorta into four quadrants with a rotation of 60 degrees. For each quadrant, the local compliance can be visualized thanks to the curve of the evolution of the surface area over time. The right image visualizes the perimeter per quadrant with different colors.

The aortic wall was partitioned into four quadrants and rotated 60 degrees to achieve orientation relative to medial, posterior, lateral, and anterior quadrants [9,12]. The different areas as well as the curve for the calculation of local compliance are displayed in Figure 8. The curve describes the surface area of the aorta over time. The in-vivo local compliance was calculated for every quadrant. Although compliance cannot be calculated ex-vivo, our hypothesis was to compare local compliance with Young’s modulus.

Moreover, the in-vivo local strain was calculated for every quadrant, allowing the comparison with the ex-vivo Young’s modulus. To calculate the local strain, the external perimeter of each quadrant was required. The right image in Figure 8 shows the external perimeter for each quadrant with different colors. Table 4 displays the average across the patients for each value.

There was no obvious link between local compliance and ex-vivo values. Instead, we observed similar behavior across in-vivo and ex-vivo measurements for the local strain.

## 4. Discussion

The aortic wall is the main structural component of the aorta. It must be elastic to expand and contract during the cardiac cycle (between systole and diastole), where the blood pressure changes rapidly. Dynamic cardiac MRI allowing the evaluation of aortic wall elasticity is a challenging imaging modality to master, particularly on a 3T magnet. Motion during the 3 Tesla MRI scan process is the leading cause of image degradation. Characteristic artifacts could be generated in the reconstructed images due to rapid or turbulent blood flow. Image blurring, ghosting, and misregistration are examples of undesirable consequences. This is especially challenging in cine CMRI, where dealing with motion caused by heartbeat remains one of the most difficult challenges. Under these circumstances, segmentation becomes a complex task because noise makes the aortic wall less visible. Besides, it is possible in the series of images to experience different noise ratios on different slices. This study aims to investigate the local elastic properties of the ascending aorta from cine-MRI. The level of the pulmonary trunk was chosen from the 2D axial MRI to localize and segment the ascending aorta with fewer artifacts and noise. Manual aorta segmentation is time-consuming and introduces intra-observer variability and bias error. Then, the segmentation process must be automated. Among the studied automatic approaches, U-Net with hyperparameter tuning performs excellently (Dice = 98.09%, IoU = 96.27%, HD = 4.88 mm) compared to the other architectures. Although Residual-U-Net has the same Dice coefficient as U-Net, the U-Net architecture has been chosen. Indeed, having the minimum Hausdorff distance makes this model lesser sensitive to outliers and miss-classifications, and the architecture is not as complex as the others. Furthermore, it is faster and uses less memory.

In Table 3, a significantly high correlation between the ground truth and predicted values of min-max aortic surfaces could be observed, confirming that our automatic segmentation provides satisfying results. Despite the high correlation, there are some overestimations with our method (36.85 and 63.13 mm${}^{2}$) for minimal and maximal surface areas compared to the ground truth. Having similar min-max areas with the ground truth is important for the compliance calculation, as the compliance formula is based on those areas. The average surface difference is low compared to the range of values ≈ 600 to 4000 mm${}^{2}$. On the other hand, for compliance, a mean error of 0.48 mm${}^{2}$/mmHg considering the range of values ≈ 0.4 to 4.3 mm${}^{2}$/mmHg becomes notable. Indeed, outliers on the min-max areas can significantly impact the calculation of compliance. Then, a lower correlation can be observed for the compliance between ground truth and calculated values.

The thickness and composition of aortic walls vary depending on their location within the organ. In this manner, we divide the aorta into four quadrants, considering the center of gravity of the aorta. Then the local compliance and strain are calculated to investigate if there is a difference in elasticity between the quadrants. Maximum and physiological Young’s modulus, local assessment of stiffness from ex-vivo tests, are compared with the local compliance of each quadrant to examine the possible association between them. Ex-vivo tests involve obtaining aorta samples during surgery and testing them in a bi-axial stretching test to determine their biomechanical characteristics. There is no apparent link between local compliance and Young’s modulus parameters in our study. Eventually, in Table 4 it can be observed that the highest compliance corresponds to the highest Young’s modulus on the lateral quadrant. This suggestion is controversial because compliance measures elasticity while maximum Young’s modulus measures the stiffness, so this comparison implies that the lateral quadrant has the highest elasticity and stiffness. According to our results, the measurement of local compliance is questionable. This may occur because we use the center of gravity to divide the aorta, then divide the area into nearly symmetrical and equal surfaces. The aorta is moving, so the center of gravity can change from one frame to another, but we do not know if it is due to the global movement of the aorta or due to the heterogeneity of the behavior of the aortic wall. Considering the previous assumptions, the measurement of local compliance may not be credible. Although, as far as we know, there is no other option for the division of a spherical object into four quadrants. Instead of compliance, an alternative solution is to calculate the local strain of each quadrant. To implement that, the local outer perimeter of each quadrant is required. Similar behavior can be observed between in-vivo strain and the ex-vivo parameters of Young’s modulus [9] (Table 4). If we compare the quadrants in pairs, medial and anterior have the minimum values, while lateral and posterior have the maximum ones. Based on these results, we can assume that high strain values correspond to high stiffness values.

The considered population in our study consisted of patients with aortic aneurysms of the ascending aorta. In future work, assessing artery stiffness for the healthy population, with subgroups according to age, sex, and cardiac risk factors is recommended to have values from matched reference groups. A study of the local elastic properties can be achieved on other arteries than the thoracic ascending aorta if the acquisition plane of the cine-MRI is perpendicular to the main axis of the artery. However, the reliability of the results depends on the spatial resolution of the image in accordance with the diameter of the artery. For example, our method can be easily used for the study of the abdominal aorta. In fenestrated endovascular repair, knowing the elastic properties of the vessel could be helpful to anticipate target vessel instability before the surgical gesture in addition to geometrical criteria [40].

## 5. Conclusions

For patients with aortic aneurysms, the elastic properties of the ascending aorta need to be considered, as they have an important impact on the health of the patient. Then, we propose an in-vivo local evaluation of aortic elastic properties from cine-MRI, knowing that the aorta introduces stiffness variability across its walls [9].

To localize and segment the aorta across a series of axial cine-MRIs, we utilize a deep learning U-Net network which performs excellent (Dice = 98.09%, IoU = 96.27%, HD = 4.88 mm). Concerning the evaluation of local elastic properties, local compliance is a questionable metric. Dividing the aorta using the center of gravity, leads to nearly symmetrical and equal surfaces for each quadrant. In advance, the center of gravity can shift from one image to another because the aorta is moving. Given the preceding assumptions, measuring local compliance may not be credible. Instead of calculating local compliance, a possible alternative is calculating each quadrant’s local strain. Comparing in-vivo strain with ex-vivo properties, we observe a possible link between in-vivo elastic properties and ex-vivo stiffness properties. This link could significantly impact medical practice, where the in-vivo evaluation of aortic elastic properties is mandatory before surgery.

Our work contributes to the investigation of the elastic properties of the aorta. More specifically we contribute to the process of making a connection between ex-vivo and in-vivo elastic properties of the ascending aorta. Our method is then compatible with clinical practice thanks to the development of a graphical user interface and an executable version of the software. Currently, the tool is only implemented in our clinical department but can be deployed in any clinical structure. In the future, assessing local aortic stiffness parameters in-vivo could be helpful to estimate the patient before surgery. Moreover, our evaluation opens the perspective of applying this comparison to other pathologies, such as Marfan’s syndrome.

## Figures and Tables

**Table 1:** Different hyperparameters tested for each U-Net architecture.

Early stopping patience	1	2	4	8	16
Starting learning rate	0.1	0.01	0.001	0.0001	
Loss function	Dice Loss	Focal Loss	Tversky Loss		
Layer Normalization	Batch	Instance	Group		

**Table 2:** Metric-based evaluation of different U-Net architectures. The best result is in bold.

Model	Dice Coeff Mean ± Std. Dev.	IoU Mean ± Std. Dev.	Hausdorff Mean ± Std. Dev. (mm)	Precision Mean ± Std. Dev.	Recall Mean ± Std. Dev.
**U-Net**	**98.09% (±0.96%)**	**96.27% (±1.83%)**	**4.88 (±1.70)**	**98.47% (±1.45%)**	97.80% (±1.74%)
Res-U-Net	**98.09% (±0.88%)**	96.26% (±1.68%)	4.91 (±1.74)	98.28% (±1.53%)	**97.99% (±1.54%)**
Att-U-Net	97.64% (±1.61%)	95.43% (±2.85%)	5.66 (±2.01)	98.62% (±1.15%)	96.83% (±3.01%)
Att-Res-U-Net	98.03% (±0.78%)	96.14% (±1.47%)	5.24 (±1.72)	98.14% (±1.27%)	97.99% (±1.64%)

**Table 3:** Comparison between the ground truth and calculated values for minimal and maximal areas, and compliance. The aortic contours are extracted with the U-Net architecture and the dataset consists of 73 patients. The second column describes how similar are the results with the ground truth, and the p-values describe if the correlation results are significant. The third column describes the average difference (and corresponding standard deviation) between in-vivo and ex-vivo results, based on Bland-Altman plots [39].

	Correlation (*p*-Value)	Mean Difference ± Std. Dev.
Minimum area	0.99 (<0.05)	36.85 mm${}^{2}$ (±52.38)
Maximum area	0.99 (<0.05)	63.13 mm${}^{2}$ (±50.22)
Compliance	0.79 (<0.05)	0.48 mm${}^{2}$/mmHg (±0.54)

**Table 4:** Comparison of in-vivo local compliance, strain, and ex-vivo Young’s modulus. Between the in-vivo dataset and the ex-vivo dataset, 22 common patients were selected. The values in bold are selected pairs with the highest values.

Quadrants	Medial	Anterior	Lateral	Posterior
**Compliance** Mean ± Std. Dev. (mm${}^{2}$/mmHg)	**0.511 (±0.25)**	0.462 (±0.19)	**0.534 (±0.21)**	0.491 (±0.18)
**In-vivo Strain** Mean ± Std. Dev.	0.086 (±0.03)	0.072 (±0.02)	**0.097 (±0.03)**	**0.095 (±0.04)**
**Maximum Young’s Modulus** Mean ± Std. Dev. (MPa) [9]	0.622 (±0.19)	0.768 (±0.24)	**1.208 (±0.47)**	**0.993 (±0.31)**
**Physiological Young’s Modulus** Mean ± Std. Dev. (MPa)	0.354 (±0.13)	0.362 (±0.15)	**0.411 (±0.13)**	**0.419 (±0.15)**

## Data Availability

Belonging to the University Hospital of Dijon (France), data cannot be shared publicly without the permission of this institution. However, they are available upon request.

## Abbreviations

The following abbreviations are used in this manuscript:

MRI	Magnetic Resonance Imaging
CMRI	Cardiac Magnetic Resonance Imaging
2D	Two Dimensional
DL	Deep Learning
IoU	Intersection over Union
SSFP	Steady State Free Precession
GRAPPA	GeneRalized Auto-calibrating Partially Parallel Acquisitions
RNN	Recurrent Neural Networks
ReLu	Rectified Linear Unit
ADAM	Adaptive Moment Estimation
TP	True Positive
FP	False Positive
FN	False Negative
